# Exercise-related changes in knee articular structures detected using magnetic resonance imaging T1ρ and T2 mapping

**DOI:** 10.1016/j.ejro.2025.100693

**Published:** 2025-10-02

**Authors:** Keita Nagawa, Hirokazu Shimizu, Saki Tsuchihashi, Kaiji Inoue, Shinji Kakemoto, Taira Shiratori, Akane Kaizu, Masahiro Koyama, Yuya Yamamoto, Masami Yoneyama, Naoki Sugita, Eito Kozawa

**Affiliations:** a Department of Radiology, Saitama Medical University, 38 Morohongou, Moroyama-machi, Iruma-gun, Saitama, Japan; b Philips Japan, Azabudai Hills Mori JP Tower 15F, 1-3-1 Azabudai, Minato-ku, Tokyo, Japan; c Department of Orthopedics, Saitama Medical University, 38 Morohongou, Moroyama-machi, Iruma-gun, Saitama, Japan

**Keywords:** Magnetic resonance image, Knee articular cartilage, Meniscus, T1ρ mapping, T2 mapping

## Abstract

**Purpose:**

To evaluate the changes in various knee joint structures before and after physical activities using magnetic resonance imaging (MRI) T1ρ and T2 mapping.

**Methods:**

MRI of the right knee was performed for 12 healthy volunteers before and after jumping rope, flexion and extension, and at rest. Different parts of articular cartilage, anterior and posterior cruciate ligaments, medial and lateral meniscus, gastrocnemius muscle and Hoffa’s fat pad were quantitatively assessed based on T1ρ and T2 values. A paired t-test was performed to determine whether the effects of various activities on different parts of the knee articular structures were statistically different.

**Results:**

The T1ρ values for the lateral meniscus decreased, while both T1ρ and T2 values for the gastrocnemius muscle increased after jumping rope. No statistically significant differences were observed in the other parts of the meniscus, cruciate ligaments, and Hoffa’s fat pad. The T1ρ and T2 values for the weight-bearing cartilages of the femur and tibia were both reduced after jumping rope. However, no statistically significant differences were observed in the cartilage after flexion and extension or at rest.

**Conclusions:**

MRI T1ρ and T2 mapping can be used to evaluate the changes in various joint structures before and after physical activities. These changes in knee tissue were hypothesized to reflect variations in tissue fluid, collagen fibers, and proteoglycan content. Further studies are required to investigate the influence of exercise on articular structures using MRI mapping techniques.

## 1 Introduction

The knee joint is a complex structure that plays a critical role in mobility and weight bearing activities. Physical activity elicits complex responses in joint tissues, including alterations in hydration, swelling, and biochemical composition of cartilage [1], [2]. Several researchers have explored the relationship between sports and changes in articular cartilage. The health of the articular cartilage is vital for maintaining joint function because it provides a low-friction surface that facilitates smooth movement while absorbing shock [3]. Some researchers believe that excessive exercise and prolonged stress on joints can have detrimental effects on joint tissues [4], [5], [6]. They have been reported to cause damage and deformation in the joints and speed up the onset and progression of osteoarthritis (OA) [4], [5], [6]. However, several studies have also suggested that moderate exercise does not increase the risk of OA in healthy people [7], [8].

Magnetic Resonance Imaging (MRI) has emerged as an essential tool for assessing joint health because of its ability to provide detailed, non-invasive images of soft tissue properties. Among various MRI techniques, T1ρ and T2 mapping have gained prominence for their ability to quantify joint tissue composition and integrity, with T1ρ reflecting the proteoglycan (PG) content, and T2 indicating the matrix of collagen fibers (CF) and water molecules [9], [10], [11]. Changes in T1ρ and T2 values can be used to evaluate the effects of exercise on articular cartilage. Previous studies have examined the acute effects of exercise and have shown that T1ρ and T2 values decrease in articular cartilage immediately after exercise [9], [12], [13]. Load and stress induce transient changes in tissue composition, primarily due to fluctuations in PG, CF, and water content [9], [12], [13]. T1ρ and T2 values are sensitive to the presence of water and the deformation of PGs and CFs, making them critical parameters for evaluating joint health post-exercise.

Many prior studies on this topic have primarily focused on investigating the effects of exercise on articular cartilage using MRI T1ρ and T2 mapping [9], [12], [13], [14]. However, whether the T1ρ and T2 values for other joint tissues, such as the meniscus and ligaments, change with physical activity has not yet been established. Prior studies have suggested that high impact and weight-bearing activities may lead to distinct changes in joint structural properties and increase the risk of OA [4], [15]. However, only a few studies have comprehensively analyzed joint structural changes in response to targeted physical exercise in healthy populations.

Therefore, this study aimed to examine the changes in the T1ρ and T2 values of different knee joint structures including articular cartilage, cruciate ligaments, meniscus, gastrocnemius muscle, and Hoffa’s fat pad before and after jumping rope, flexion and extension, and rest. This study aimed to gain a better understanding of the responses of the joint tissues to various dynamic activities by evaluating their differences. The results of this study will help develop effective strategies in sports medicine, rehabilitation, and injury prevention and guide recommendations for training to optimize joint health and performance.

## 2 Methods

### 2.1 Participants and image acquisitions

Fourteen healthy volunteers aged between 20 and 40 years who provided written informed consent were recruited. The exclusion criteria were as follows: patients with a history of injury, treatment, or surgery of the knee joint; those with any symptoms or abnormalities noted in the knee joint; those who were prohibited from or unable to participate in sports or exercise for any reason; those who were contraindicated for MRI; those who were prone to MRI artifacts and had difficulty in measuring signal levels; those with a history of malignancy; and those who were pregnant. Of the 13 volunteers, one developed back pain while jumping rope and withdrew from the study. Therefore, 12 volunteers (nine males and three females) with an average age of 26 were finally included.

MRI scans of the right knees of the participants were obtained before and after jumping rope (10 sets of 30 jump ropes with an interval of 15–30 s between each set). One week later, MRI scans were obtained before and after flexion and extension (10 sets of 20 flexion and extension exercises, with 15–30 s between each set). Two weeks later, the same MRI scans were performed before and after rest (30 min of bed rest). A 30-minute rest was allowed before the first MRI scan of each examination to reduce the effects of previous activity on the joint tissue.

**Fig. 1:**
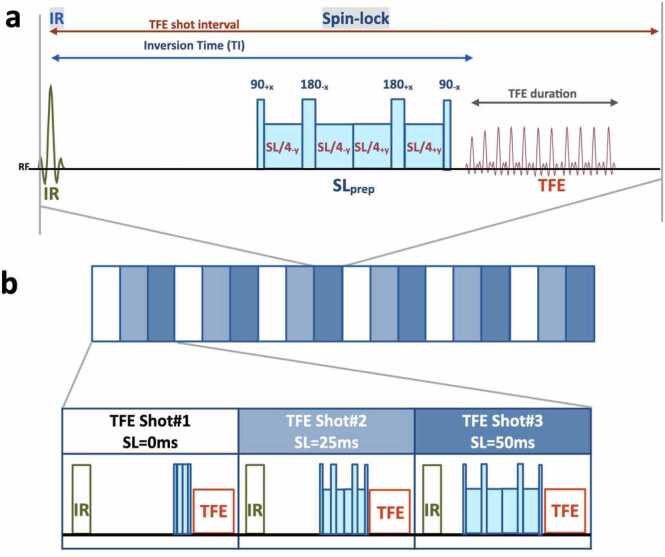
Schematic overview of the sequence for 3-dimentional isotropic T1ρ-mapping. (**a**) The sequence is based on an inversion-recovery and double-refocusing spin-lock (SL) prepared segmented gradient echo (turbo field-echo: TFE), with ProSet water-selective excitation. Inversion-recovery was applied for suppression of synovial fluid. (**b**) Three images with different SL preparation times (SL = 0, 25, and 50 ms) were acquired with interleaved acquisition. Amplitude of the SL pulse was set to 500 Hz.

**Fig. 2:**
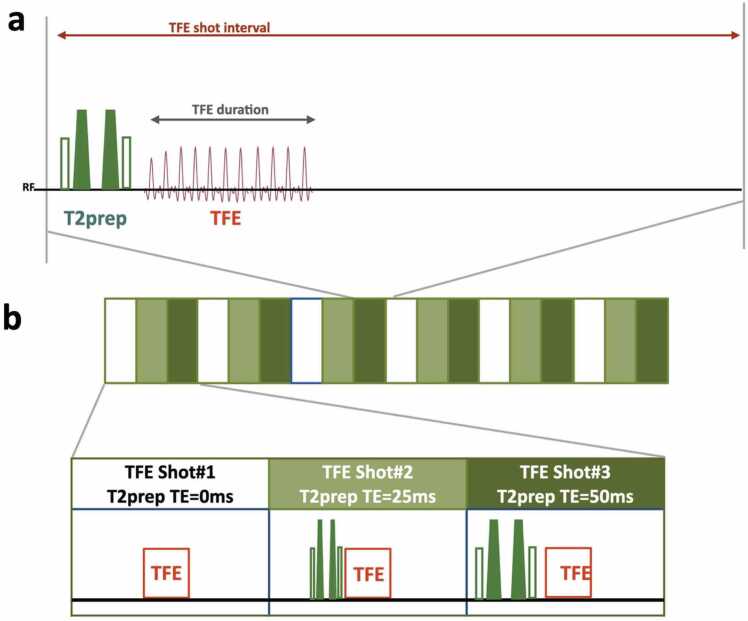
Scheme of the sequence for 3-dimentional isotropic T2-mapping. (**a**) The T2-mapping sequence is based on T2ρ-prepared turbo field-echo (TFE). (**b**) Three images with different T2-preparation times (T2prep TE = 0, 25, and 50 ms) were acquired with interleaved acquisition.

All MRI scans were performed using a 3.0-T system (Ingenia Elition; Philips Healthcare, Netherlands) with a vendor-specific 16-channel knee coil. The 3-dimentional isotropic T1ρ-mapping is a technique based on inversion-recovery and double-refocusing spin-lock (SL) prepared segmented gradient echo (turbo field-echo: TFE) with ProSet water-selective excitation. In the present study, inversion-recovery was applied for suppression of the synovial fluid. Three images with different SL preparation times (SL = 0, 25, and 50 ms) were acquired using interleaved acquisition. Amplitude of the SL pulse was set to 500 Hz. The TFE shot interval was used as a repetition time (TR) for T1ρ calculation instead of the TE of the readout TFE sequence itself. Conversely, T2-mapping is a technique based on T2ρ-prepared TFE sequences. In the present study, three images with different T2-preparation times (T2prep echo time (TE) = 0, 25, and 50 ms) were acquired using interleaved acquisition. For T2 calculation, the TFE shot interval and T2prep TE were used as TR and TE, respectively, instead of the TR and TE of the readout TFE sequence. Schemes of the sequences for 3-dimensional isotropic T1ρ-mapping and T2-mapping are presented in Fig. 1, Fig. 2, respectively.

The imaging protocols were as follows:

3-dimentional T1ρ-weighted images: TR 6000 ms for T1ρ fitting and 8.5 ms for TFE readout; TE 3.5 ms; flip angle 35°; number of averages 1; number of slices 200; slice thickness 1 mm; field of view 14 cm; matrix 140 × 140; spin lock time 0, 25, 50 ms; spin lock frequency 500 Hz)

3-dimensional T2 mapping images: TR 2000 ms for T2 fitting and 4.5 ms for TFE readout; TE 0, 25, 50 ms for T2 fitting and 1.9 ms for TFE readout; flip angle 35°; number of averages 1; number of slices 200; slice thickness 1 mm; field of view 14 cm; matrix 140 × 140; echo train length 64.

The T1ρ and T2 maps were computed on a pixel-by-pixel basis using a mono-exponential decay model, as follows:

$$M\left(\mathrm{SLT}\right)=M0\exp\left(-\frac{\mathrm{SLT}}{T1\rho }\right)$$

Where M0 and M (SLT) denote the equilibrium magnetization and T1ρ-prepared magnetization with the spin-lock time (SLT), respectively.

$$M\left(\mathrm{TE}\right)=M0\exp\left(-\frac{\mathrm{TE}}{T_{2}}\right)$$

Where M0 and M (TE) denote the equilibrium magnetization and T2 magnetization with the echo time (TE), respectively.

Both maps were generated by fitting the intensity of each pixel using a non-negative least-square fitting method.

### 2.2 Measurements of T1ρ and T2 values of knee joint structures

**Fig. 3:**
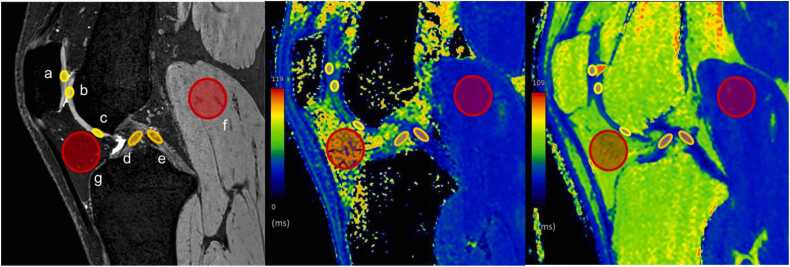
An example of the delineated region-of-interest on the knee articular tissues in the intercondylar plane of a T2*-weighted fast-field echo image (left), a T1ρ-mapping image (center), and a T2-mapping image (right). Ovoid regions-of-interest were placed on the patellar cartilage (**a**); the anterior (**b**) and anteroinferior (**c**) cartilages of the femur; the centers of the anterior (**d**) and posterior (**e**) cruciate ligaments; gastrocnemius muscle (**f**); and Hoffa's fat pad (**g**).

**Fig. 4:**
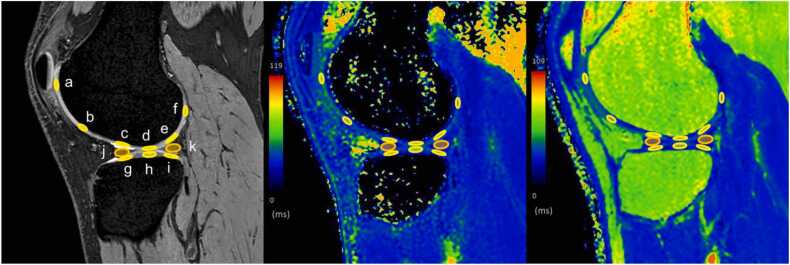
An example of the delineated region-of-interest on the knee articular tissues in the medial plane of a T2*-weighted fast-field echo image (left), a T1ρ mapping image (center), and a T2 mapping image (right). Ovoid regions-of-interest were placed on the anterior weight-bearing (AWB) (**a**), weight-bearing (WB) (**b**), posterior weight-bearing (PWB) (**c**), and posterior (**d**) cartilages of the femur; the AWB (**e**), WB (**f**), and PWB (**g**) cartilages of the tibia; and anterior (**h**) and posterior (**i**) segments of the medial meniscus.

**Fig. 5:**
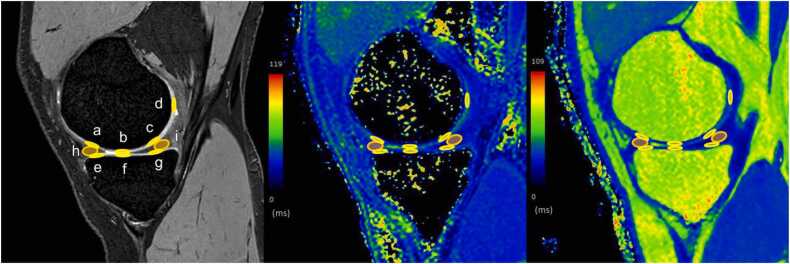
An example of the delineated region-of-interest on the knee articular tissues in the lateral plane of a T2*-weighted fast-field echo image (left), a T1ρ mapping image (center), and a T2 mapping image right). Ovoid regions-of-interest were placed on the anterior (**a**), anteroinferior (**b**), anterior weight-bearing (AWB) (**c**), weight-bearing (WB) (**d**), posterior weight-bearing (PWB) (**e**), and posterior (**f**) cartilages of the femur; the AWB (**g**), WB (**h**), and PWB (**i**) cartilages of the tibia; and the anterior (**j**) and posterior (**k**) segments of the lateral meniscus.

Two radiologists with eight and seven years of experience (K.N. and H.S.) who were blinded to the clinical information independently delineated region of interest (ROI) and subsequently reached a consensus. The areas of the following parts of the knee articular structures were manually delineated after the MR images were loaded into the workstation Synapse VINCENT version 6.8 (Fuji Film, Tokyo, Japan). In the intercondylar slice (slice with the centers of the anterior cruciate ligament [ACL] and posterior cruciate ligament [PCL] imaged), ovoid ROIs were placed on the patellar cartilage, anterior and anteroinferior cartilage of the femur, centers of the ACL and PCL, gastrocnemius muscle, and Hoffa's fat pad (Fig. 3). In the medial slice (slice through the free edge of the medial meniscus body), ovoid ROIs were placed on the anterior weight-bearing (AWB), weight-bearing (WB), posterior weight-bearing (PWB) and posterior cartilage of the femur; the AWB, WB and PWB cartilage of the tibia; and the anterior and posterior segments of medial meniscus (Fig. 4). In the lateral slice (slice through the free edge of the lateral meniscus body), ovoid ROIs were placed on the anterior, anteroinferior, AWB, WB, PWB and posterior cartilage of the femur; the AWB, WB and PWB cartilage of the tibia; and the anterior and posterior segments of the lateral meniscus (Fig. 5). The delineated ROI has an area of 5 mm^2^ for the cartilage, 10 mm^2^ for the ligaments and meniscus, and 100 mm^2^ for the muscle and fat pads.

### 2.3 Statistical analysis

Statistical analysis was performed using SPSS version 29.0 (IBM Corporation, Armonk, NY, USA) at a significance level of P < 0.05. The data are presented as the means ± standard deviations. The intraclass correlation coefficient (ICC) was calculated to evaluate the interobserver reproducibility in the segmentation process. A paired *t*-test was performed to determine whether the influences of jumping rope, flexion and extension, and rest on different parts of the knee articular structures were statistically different.

## 3 Results

The ICC values of all T1ρ and T2 mapping data are summarized in Table 1, Table 2. Excellent interobserver reproducibility was observed for all statistical values.

**Table 1:** Intraclass correlation coefficients for evaluating the inter-observer reproducibility of the segmentation process of T1ρ mapping imaging.

Intercondylar part	Cartilage			Ligaments		Muscle	Fat pad
	Patellar cartilage	Femoral anterior cartilage	Femoral anteroinferior cartilage	Anterior cruciate ligament	Posterior cruciate ligament	Gastrocnemius muscle	Hoffa's fat pad
	0.930	0.934	0.916	0.920	0.943	0.927	0.977
Medial part	Cartilage						
	Femoral anterior weight-bearing cartilage	Femoral weight-bearing cartilage	Femoral posterior weight-bearing cartilage	Femoral posterior cartilage	Tibial anterior weight-bearing cartilage		
	0.795	0.847	0.907	0.846	0.926		
	Cartilage		Meniscus				
	Tibial weight-bearing cartilage	Tibial posterior weight-bearing cartilage	Anterior segment of medial meniscus	Posterior segment of medial meniscus			
	0.941	0.956	0.840	0.944			
Lateral part	Cartilage						
	Femoral anterior cartilage	Femoral anteroinferior cartilage	Femoral anterior weight-bearing cartilage	Femoral weight-bearing cartilage	Femoral posterior weight-bearing cartilage	Femoral posterior cartilage	
	0.983	0.929	0.882	0.963	0.951	0.901	
	Cartilage			Meniscus			
	Tibial anterior weight-bearing cartilage	Tibial weight-bearing cartilage	Tibial posterior weight-bearing cartilage	Anterior segment of lateral meniscus	Posterior segment of lateral meniscus		
	0.914	0.960	0.924	0.963	0.837

**Table 2:** Intraclass correlation coefficients for evaluating the inter-observer reproducibility of the segmentation process of T2 mapping imaging.

Intercondylar part	Cartilage			Ligaments		Muscle	Fat pad
	Patellar cartilage	Femoral anterior cartilage	Femoral anteroinferior cartilage	Anterior cruciate ligament	Posterior cruciate ligament	Gastrocnemius muscle	Hoffa's fat pad
	0.790	0.935	0.932	0.972	0.905	0.967	0.795
Medial part	Cartilage						
	Femoral anterior weight-bearing cartilage	Femoral weight-bearing cartilage	Femoral posterior weight-bearing cartilage	Femoral posterior cartilage	Tibial anterior weight-bearing cartilage		
	0.978	0.939	0.889	0.962	0.932		
	Cartilage		Meniscus				
	Tibial weight-bearing cartilage	Tibial posterior weight-bearing cartilage	Anterior segment of medial meniscus	Posterior segment of medial meniscus			
	0.921	0.857	0.978	0.807			
Lateral part	Cartilage						
	Femoral anterior cartilage	Femoral anteroinferior cartilage	Femoral anterior weight-bearing cartilage	Femoral weight-bearing cartilage	Femoral posterior weight-bearing cartilage	Femoral posterior cartilage	
	0.960	0.885	0.907	0.893	0.906	0.943	
	Cartilage			Meniscus			
	Tibial anterior weight-bearing cartilage	Tibial weight-bearing cartilage	Tibial posterior weight-bearing cartilage	Anterior segment of lateral meniscus	Posterior segment of lateral meniscus		
	0.913	0.966	0.978	0.756	0.827		

In the intercondylar region (Tables 3 and 4), no significant differences were observed in the cartilage, cruciate ligaments, or Hoffa’s fat pad. For the gastrocnemius muscle, both T1ρ and T2 values increased after jumping rope with a statistical difference.

**Table 3:** Summary of exercise-related changes in T1ρ values of the knee joint tissues in the intercondylar plane.

	Jumping rope			Flexion/extension			Rest		
	Before	After	P-value	Before	After	P-value	Before	After	P-value
Cartilage									
Patellar cartilage	43.1 ± 2.6	42.2 ± 2.7	0.124	43.1 ± 1.7	42.0 ± 2.2	0.118	42.7 ± 2.1	42.4 ± 2.9	0.521
Femoral anterior cartilage	48.4 ± 2.6	47.5 ± 2.5	0.096	49.0 ± 2.0	47.9 ± 2.6	0.201	48.9 ± 3.0	49.3 ± 2.3	0.496
Femoral anteroinferior cartilage	41.9 ± 4.1	40.1 ± 2.8	0.142	42.8 ± 4.7	41.2 ± 3.8	0.114	41.9 ± 3.0	42.2 ± 3.2	0.663
Ligaments									
Anterior cruciate ligament	39.7 ± 4.7	40.5 ± 3.9	0.363	39.3 ± 3.7	39.4 ± 3.8	0.934	38.0 ± 3.2	38.7 ± 3.2	0.391
Posterior cruciate ligament	29.3 ± 2.4	29.0 ± 2.6	0.732	30.0 ± 2.8	29.1 ± 2.8	0.487	29.1 ± 2.2	29.1 ± 2.5	0.979
Gastrocnemius muscle	30.1 ± 1.5	35.1 ± 3.8	**0.011**	31.2 ± 1.5	30.8 ± 2.8	0.695	30.4 ± 2.0	31.1 ± 1.6	0.329
Hoffa's fat pad	65.2 ± 8.0	64.9 ± 7.9	0.813	63.6 ± 7.6	60.9 ± 8.2	0.155	64.6 ± 7.2	65.9 ± 7.3	0.463

Data are presented as the mean ± standard deviation. The unit is milliseconds. Paired t-tests were performed on the values before and after jumping rope, flexion/extension, and resting.

**Table 4:** Summary of exercise-related changes in T2 values of the knee joint tissues in the intercondylar plane.

	Jumping rope			Flexion/extension			Rest		
	Before	After	P-value	Before	After	P-value	Before	After	P-value
Cartilage									
Patellar cartilage	30.0 ± 1.6	30.4 ± 1.6	0.344	30.8 ± 1.1	31.2 ± 1.3	0.226	30.1 ± 1.5	30.2 ± 1.4	0.595
Femoral anterior cartilage	36.7 ± 3.3	37.1 ± 2.8	0.330	38.0 ± 3.4	37.5 ± 3.8	0.310	36.5 ± 3.0	36.6 ± 3.2	0.780
Femoral anteroinferior cartilage	29.8 ± 2.4	29.2 ± 2.0	0.167	29.6 ± 3.3	28.6 ± 3.1	0.208	30.1 ± 3.9	29.7 ± 4.0	0.508
Ligaments									
Anterior cruciate ligament	29.6 ± 3.6	29.8 ± 3.2	0.764	28.6 ± 3.8	27.9 ± 3.0	0.411	27.8 ± 3.7	28.2 ± 4.2	0.423
Posterior cruciate ligament	21.5 ± 1.0	21.9 ± 2.1	0.541	22.3 ± 1.9	22.3 ± 2.8	0.944	22.0 ± 1.2	21.9 ± 1.5	0.832
Gastrocnemius muscle	29.2 ± 1.5	32.6 ± 1.9	**< 0.001**	29.5 ± 1.6	29.6 ± 1.4	0.755	29.5 ± 1.7	29.9 ± 1.2	0.274
Hoffa's fat pad	62.1 ± 2.4	63.0 ± 2.1	0.155	61.0 ± 2.4	62.0 ± 2.2	0.105	61.3 ± 1.9	60.9 ± 2.3	0.310

Data are presented as the mean ± standard deviation. The unit is milliseconds. Paired t-tests were performed on the values before and after jumping rope, flexion/extension, and resting.

In the medial region (Table 5, Table 6), both T1ρ and T2 values decreased for the weight-bearing cartilages of the femur and tibia after jumping rope with a statistical difference.

**Table 5:** Summary of exercise-related changes in T1ρ values of the knee joint tissues in the medial plane.

	Jumping rope			Flexion/extension			Rest		
	Before	After	P-value	Before	After	P-value	Before	After	P-value
Cartilage									
Femoral anterior weight-bearing cartilage	40.6 ± 3.6	39.4 ± 3.1	0.184	41.4 ± 3.3	40.8 ± 2.8	0.170	39.8 ± 3.5	39.8 ± 3.4	0.951
Femoral weight-bearing cartilage	39.0 ± 2.7	37.8 ± 2.7	0.161	38.4 ± 4.1	37.2 ± 3.6	0.210	38.1 ± 2.7	37.7 ± 3.0	0.631
Femoral posterior weight-bearing cartilage	42.7 ± 3.0	41.4 ± 4.2	0.149	41.2 ± 2.1	42.6 ± 3.4	0.112	44.0 ± 3.5	44.0 ± 4.0	0.992
Femoral posterior cartilage	45.8 ± 1.8	44.2 ± 2.7	0.103	46.4 ± 3.2	44.0 ± 4.8	0.119	44.9 ± 2.6	46.5 ± 4.1	0.189
Tibial anterior weight-bearing cartilage	38.7 ± 4.0	37.1 ± 3.3	0.084	39.1 ± 2.9	38.0 ± 2.1	0.225	38.3 ± 2.4	37.4 ± 3.7	0.400
Tibial weight-bearing cartilage	37.8 ± 4.1	35.7 ± 4.5	**0.012**	37.5 ± 4.3	36.3 ± 2.8	0.105	36.3 ± 2.4	37.3 ± 3.4	0.173
Tibial posterior weight-bearing cartilage	37.5 ± 3.2	36.2 ± 3.2	**0.034**	37.0 ± 3.3	36.4 ± 3.3	0.185	37.0 ± 3.5	37.3 ± 3.0	0.622
Meniscus									
Anterior segment of medial meniscus	28.4 ± 4.1	26.5 ± 2.1	0.185	27.8 ± 2.7	26.8 ± 2.4	0.288	28.4 ± 2.8	27.9 ± 2.1	0.427
Posterior segment of medial meniscus	23.6 ± 1.5	22.2 ± 1.7	0.063	23.0 ± 1.9	22.2 ± 1.9	0.141	23.4 ± 1.6	24.0 ± 1.7	0.364

Data are presented as the mean ± standard deviation. The unit is milliseconds. Paired t-tests were performed on the values before and after jumping rope, flexion/extension, and resting.

**Table 6:** Summary of exercise-related changes in T2 values of the knee joint tissues in the medial plane.

	Jumping rope			Flexion/extension			Rest		
	Before	After	P-value	Before	After	P-value	Before	After	P-value
Cartilage									
Femoral anterior weight-bearing cartilage	28.0 ± 3.6	27.9 ± 3.4	0.825	28.1 ± 4.3	27.9 ± 2.8	0.854	27.6 ± 4.1	28.1 ± 4.6	0.321
Femoral weight-bearing cartilage	26.4 ± 4.7	25.5 ± 3.8	0.230	25.3 ± 3.3	24.5 ± 4.1	0.400	24.8 ± 3.1	25.6 ± 3.7	0.143
Femoral posterior weight-bearing cartilage	36.8 ± 2.9	35.5 ± 3.5	0.116	36.2 ± 4.0	34.6 ± 4.8	0.167	35.9 ± 3.6	36.4 ± 3.4	0.432
Femoral posterior cartilage	36.1 ± 3.7	35.2 ± 3.7	0.151	35.4 ± 2.5	34.8 ± 3.0	0.198	35.0 ± 2.6	35.1 ± 3.0	0.730
Tibial anterior weight-bearing cartilage	31.8 ± 3.9	30.5 ± 3.9	0.057	31.5 ± 3.9	30.5 ± 3.9	0.155	31.7 ± 3.4	32.4 ± 3.6	0.334
Tibial weight-bearing cartilage	31.1 ± 4.8	28.1 ± 3.1	**0.013**	31.1 ± 6.1	29.2 ± 3.5	0.150	29.8 ± 3.6	30.5 ± 3.0	0.351
Tibial posterior weight-bearing cartilage	32.6 ± 3.9	30.4 ± 2.6	0.073	32.3 ± 3.4	31.1 ± 3.1	0.198	30.5 ± 2.6	30.6 ± 2.7	0.795
Meniscus									
Anterior segment of medial meniscus	20.1 ± 1.7	20.8 ± 2.4	0.335	19.6 ± 1.7	20.0 ± 2.4	0.587	19.9 ± 2.0	20.1 ± 1.3	0.657
Posterior segment of medial meniscus	19.9 ± 1.2	20.0 ± 1.1	0.770	19.3 ± 1.1	19.6 ± 1.0	0.205	18.5 ± 1.9	18.8 ± 1.4	0.552

Data are presented as the mean ± standard deviation. The unit is milliseconds. Paired t-tests were performed on the values before and after jumping rope, flexion/extension, and resting.

In the lateral region (Table 7, Table 8), the T1ρ and T2 values decreased for the femoral anterior cartilage, as well as the femoral and tibial weight-bearing cartilages, after jumping rope with a statistical difference. For the meniscus, the T1ρ value decreased for the anterior segment of the lateral meniscus after jumping rope. No statistically significant difference was observed in the joint tissues after flexion or extension and at rest.

**Table 7:** Summary of exercise-related changes in T1ρ values of the knee joint tissues in the lateral plane.

	Jumping rope			Flexion/extension			Rest		
	Before	After	P-value	Before	After	P-value	Before	After	P-value
Cartilage									
Femoral anterior cartilage	51.5 ± 3.9	50.3 ± 4.1	**0.010**	51.6 ± 4.5	50.5 ± 3.3	0.182	51.5 ± 4.1	51.9 ± 3.7	0.522
Femoral anteroinferior cartilage	45.0 ± 3.1	43.7 ± 3.4	**0.015**	44.8 ± 3.0	43.9 ± 3.5	0.123	43.4 ± 4.2	44.1 ± 4.3	0.184
Femoral anterior weight-bearing cartilage	38.5 ± 4.2	35.7 ± 2.4	**0.046**	39.0 ± 3.6	37.8 ± 3.3	0.125	37.9 ± 4.7	38.2 ± 4.5	0.553
Femoral weight-bearing cartilage	37.3 ± 4.9	34.9 ± 3.7	0.080	36.0 ± 3.3	34.6 ± 3.9	0.135	35.7 ± 4.3	35.8 ± 3.4	0.837
Femoral posterior weight-bearing cartilage	43.3 ± 2.8	41.9 ± 3.9	0.116	42.3 ± 1.8	41.3 ± 2.2	0.140	41.5 ± 1.8	41.3 ± 3.7	0.872
Femoral posterior cartilage	42.6 ± 2.5	40.7 ± 3.8	0.110	43.0 ± 2.9	41.9 ± 3.6	0.296	43.7 ± 2.5	43.2 ± 4.1	0.599
Tibial anterior weight-bearing cartilage	40.8 ± 4.2	37.1 ± 5.8	**0.040**	39.5 ± 5.1	37.8 ± 2.8	0.113	38.0 ± 5.2	38.6 ± 4.6	0.258
Tibial weight-bearing cartilage	32.2 ± 1.7	30.5 ± 2.8	**0.030**	32.2 ± 3.1	30.8 ± 2.5	0.169	32.6 ± 3.3	33.0 ± 3.8	0.504
Tibial posterior weight-bearing cartilage	31.9 ± 3.4	29.7 ± 4.1	**0.041**	31.9 ± 5.9	30.7 ± 4.8	0.150	31.3 ± 2.7	31.7 ± 2.8	0.409
Meniscus									
Anterior segment of lateral meniscus	28.9 ± 3.6	26.3 ± 2.0	**0.048**	28.4 ± 3.1	27.2 ± 3.0	0.079	27.3 ± 1.2	27.5 ± 2.7	0.797
Posterior segment of lateral meniscus	24.7 ± 2.8	23.4 ± 2.1	**0.046**	24.8 ± 1.2	24.0 ± 2.0	0.108	24.0 ± 1.7	23.5 ± 2.5	0.382

Data are presented as the mean ± standard deviation. The unit is milliseconds. Paired t-tests were performed on the values before and after jumping rope, flexion/extension, and resting.

**Table 8:** Summary of exercise-related changes in T2 values of the knee joint tissues in the lateral plane.

	Jumping rope			Flexion/extension			Rest		
	Before	After	P-value	Before	After	P-value	Before	After	P-value
Cartilage									
Femoral anterior cartilage	37.3 ± 3.1	35.6 ± 3.1	**0.018**	37.0 ± 4.1	36.9 ± 3.9	0.141	37.5 ± 3.3	37.3 ± 3.2	0.404
Femoral anteroinferior cartilage	32.1 ± 2.7	30.2 ± 2.6	**0.018**	32.5 ± 2.8	31.0 ± 1.8	0.114	31.2 ± 3.1	31.2 ± 3.1	0.875
Femoral anterior weight-bearing cartilage	27.4 ± 3.5	25.4 ± 4.7	**0.039**	28.1 ± 3.2	25.8 ± 3.0	0.111	27.1 ± 2.8	27.4 ± 3.2	0.622
Femoral weight-bearing cartilage	27.1 ± 3.7	24.8 ± 3.4	**0.010**	26.5 ± 3.8	25.2 ± 2.8	0.107	25.4 ± 2.4	25.7 ± 2.6	0.511
Femoral posterior weight-bearing cartilage	37.3 ± 2.2	36.3 ± 2.6	0.118	37.4 ± 2.2	36.6 ± 1.9	0.158	37.2 ± 2.7	37.0 ± 3.6	0.896
Femoral posterior cartilage	33.0 ± 2.3	31.9 ± 2.6	0.091	32.5 ± 2.3	31.5 ± 2.7	0.314	32.3 ± 1.8	32.4 ± 1.9	0.896
Tibial anterior weight-bearing cartilage	28.7 ± 4.4	26.9 ± 3.4	0.096	28.5 ± 2.6	26.9 ± 2.8	0.101	27.6 ± 2.9	27.5 ± 3.2	0.827
Tibial weight-bearing cartilage	25.3 ± 4.4	23.6 ± 3.3	**0.037**	24.8 ± 3.9	24.0 ± 3.4	0.081	24.3 ± 3.3	23.8 ± 3.6	0.325
Tibial posterior weight-bearing cartilage	23.9 ± 4.9	22.0 ± 4.3	0.052	23.6 ± 4.5	24.1 ± 4.4	0.589	24.8 ± 5.7	24.4 ± 4.3	0.618

Data are presented as the mean ± standard deviation. The unit is milliseconds. Paired t-tests were performed on the values before and after jumping rope, flexion/extension, and resting.

## 4 Discussion

In the present study, we evaluated the changes in various knee joint structures before and after exercise using MRI T1ρ and T2 mapping. Overall, we found that the T1ρ value for the lateral meniscus decreased after exercise, while both the T1ρ and T2 values for the gastrocnemius muscle increased after jumping rope. No statistically significant differences were observed in any other parts, including the meniscus, cruciate ligaments, and Hoffa’s fat pad. We also observed that both the T1ρ and T2 values decreased for the weight-bearing cartilages of the femur and tibia after jumping rope.

The relationship between the physiological activity and structural changes in the knee joint has been previously reported. Load and stress induce biochemical changes in the joint tissues, including shifts in water content within the matrix, deformation of CF, and relative increases in PG content. Most prior studies in this field have tended to consider the effects of exercise on articular cartilage using MRI T1ρ and T2 mapping [9], [12], [13], [14]. The structural changes in the cartilage matrix should be reflected as a decrease in both T1ρ and T2 values, because T1ρ values are negatively correlated with PG content and T2 values are positively correlated with water and CF content. Our study confirmed that both T1ρ and T2 values for the load-bearing cartilage decreased after physiological activity, as the previous results have shown. However, studies concerning changes in T1ρ and T2 values in the other joint tissues associated with physical activity, such as the meniscus and ligaments, have been limited. Considering the role these structures play in supporting the knee, it would be beneficial to investigate these physiological changes using MRI T1-weighted and T2 mapping.

Regarding the meniscus, we found that T1ρ values in the lateral meniscus decreased after completing the jumping exercise. However, the relationship between physical activity and the MRI signal changes in the meniscus remains controversial. Kursunoglu-Brahme et al. [16] observed increased meniscal signal intensity on MRI after yoga, although the clinical significance of these findings is uncertain. Krampla et al. [17] reported minor meniscal signal changes that were noticeable immediately after running but returned to normal 6 weeks after the competition. Furthermore, Stehling et al. [18] measured T1ρ and T2 values of the meniscus in marathon runners and reported that they increased significantly after the competition, followed by a decrease in T2 values, while the T1ρ values remained high. This seems to contradict our results, but the most important point is that their measurements were taken 48–72 h after the marathon and not immediately after the race [18]. Our hypothesis was that relaxation time is reduced immediately after loading exercises. This may be attributed to transient fluctuations in PG, CF, and water content, similar to those described above for knee articular cartilage.

Overall, our results revealed no statistically significant differences in any other parts of the meniscus, cruciate ligaments, or Hoffa’s fat pad. Some studies have suggested that dynamic flexion induces stress and strain on the cruciate ligaments [24], [25]. However, other studies have reported that these effects are limited and not significant [26]. One prior study [27] investigated the seasonal physiological changes in the ACL of alpine skiers using T2* mapping. This study showed that the T2* value of the anterior cruciate ligament increased during the peak ski season (January to April), and decreased thereafter (July) [27]. The increase in ACL T2* value has therefore been considered to reflect a decrease in cell density, collagen organization, and tensile loading [27]. For the Hoffa’s fat pad, changes in quantitative MRI measurements were assessed in marathon runners [28]. After the marathon, the T1 and T2 values of the Hoffa's fat pad decreased, while fat fraction increased [28]. This indicates an increase in the proportion of adipose tissue within the Hoffa's fat pad [28]. Based on these prior studies, the T1ρ and T2 value changes in the ligaments and fat pad following exercise can be anticipated; however, no significant differences were observed in this study. Therefore, further verification is required, with sufficient exercise load and a large sample size, in order to evaluate significant changes.

For the gastrocnemius muscle, we found that both T1ρ and T2 values increased after jumping rope. The relaxation times of muscles can fluctuate due to several factors. For example, changes in muscular composition, such as muscle fiber type, may influence muscle relaxation time [19]. In muscular fibrosis, damaged skeletal muscle is replaced by excessive CFs [20], [21], resulting in increases in the T1ρ and T2 values. However, these fibrotic changes are considered chronic, whereas acute changes in the skeletal muscle primarily include increased water content and microperfusion. Iijima et al. [22] previously reported an increase in the T2 values of the rotator cuff muscles with increasing tear extent. Maillard et al. [23] previously evaluated the utility of T2 values as quantitative measures of muscle inflammation. However, there is currently no research on changes in the relaxation times of the skeletal muscles after exercise. Based on the present results, we hypothesize that increased T1ρ and T2 values may be caused by transient damage to the striated skeletal muscle and increased water content.

In our study, the T1ρ and T2 values of cartilage differed for the knee articular regions; both T1ρ and T2 values decreased for the load-bearing cartilages in the lateral and medial regions of the femur and tibia after exercise, but remained unchanged for other cartilage regions. Loaded cartilage may exert greater pressure and friction, and is considered more vulnerable to damage during exercise. A prior study investigating the stress characteristics of the load-bearing cartilage showed that the posterior portion was more dominant than the anterior portion [13]; however, this was not evident in our results. Indeed, our results showed that both the T1ρ and T2 values for the femoral anterior cartilage (patellofemoral cartilage) in the lateral region decreased after exercise. The lateral portion of the patellofemoral cartilage is the primary contact area, and is therefore more susceptible to injury than the medial portion during physical activity [13]. However, in our results, no significant differences were observed in the T1ρ and T2 values of the patellofemoral and trochlear cartilages in the intercondylar region.

In the present study, changes in the T1ρ and T2 values for the knee articular cartilage were more conspicuous after jumping rope than after flexion and extension. Chen et al. [13] previously evaluated the differences in T1ρ and T2 values of the knee articular cartilage after different activities observing the following trend of cartilage changes during exercise: stair climbing > running > walking [13]. However, they did not consider jumping or bending, but suggested that the excessive stress of jumping rope could cause more pronounced changes in the cartilage than flexion and extension. Further, biomechanical analysis should be incorporated to strictly investigate the loading of the knee joint tissues. By comparing these results, we were able to understand the exact relationship between the actual force applied to the joint and MRI signal change.

Overall, this study focused on changes in knee joint structure immediately after exercise. These acute changes are particularly important in injury prevention as they reveal what types of load and stress lead to joint damage. Thus, our findings provide a framework of joint tissue responses to loads and stresses, and could have clinical utility when providing exercise advice to patients. At the same time, the delayed effects of exercise on joint structures are also useful as they may help to predict structural recovery following appropriate exercise and guide the optimization of rehabilitation strategies. Notably, some prior studies have dealt with these delayed effects; for example, one study showed that 3-months of moderate exercise reduced T1ρ values in the high-stress areas of the knee in patients with early osteoarthritis, indicating a higher PG content and better overall cartilage health. However, this study could not evaluate the delayed effects of exercise. This is a notable limitation of our study; if this could be evaluated, the results will likely be more clinically useful.

The present study had several limitations. Firstly, the sample was too small to perform adequate statistical evaluations. A larger sample is required to increase confidence. In addition, the lack of diversity in exercise also limits the conclusions, as conducting a broader range of physical activities at different intensities could allow the elucidation of more detailed correlations between MRI biomarkers and activity intensities. Further, elliptical ROIs were chosen in this study for technical reasons; however, for precise evaluation, the entire area of each joint structure should ideally be evaluated. Furthermore, the biomechanical mechanisms of the knee joint were not sufficiently considered. The loading forces or stress levels applied to the joint should be calculated and correlated with our results because they are important factors for understanding the changes in joint structures during exercise. Strictly speaking, comparisons of T1ρ and T2 value changes across various activities are difficult without objective measurements. This study also did not correlate the results with any histopathological analyses as the gold standard. These issues should be addressed in future studies.

In conclusion, the present study evaluated the changes in various knee joint structures before and after exercise using MRI T1ρ and T2 mapping. Overall, we found that the T1ρ value for the lateral meniscus decreased after exercise, while both the T1ρ and T2 values for the gastrocnemius muscle increased after jumping rope. We observed no significant differences in the other parts of the meniscus, cruciate ligaments, and Hoffa’s fat pad. Various joint tissue responses to loads and stresses could be evaluated by MRI T1ρ and T2 mapping and should be further investigated in future studies.

## CRediT authorship contribution statement

**Yuya Yamamoto:** Writing – original draft, Data curation, Conceptualization. **Masami Yoneyama:** Writing – original draft, Methodology, Investigation, Formal analysis, Data curation. **Naoki Sugita:** Writing – original draft, Supervision, Resources, Formal analysis, Conceptualization. **Eito Kozawa:** Writing – review & editing, Writing – original draft, Supervision, Data curation, Conceptualization. **Keita Nagawa:** Writing – review & editing, Writing – original draft, Validation, Supervision, Software, Project administration, Methodology, Investigation, Funding acquisition, Formal analysis, Data curation, Conceptualization. **Hirokazu Shimizu:** Writing – original draft, Project administration, Methodology, Investigation, Formal analysis, Data curation, Conceptualization. **Saki Tsuchihashi:** Writing – original draft, Project administration, Methodology, Investigation, Formal analysis, Data curation, Conceptualization. **Kaiji Inoue:** Writing – review & editing, Writing – original draft, Supervision, Project administration, Methodology, Formal analysis, Data curation, Conceptualization. **Shinji Kakemoto:** Writing – original draft, Data curation, Conceptualization. **Taira Shiratori:** Writing – original draft, Data curation, Conceptualization. **Akane Kaizu:** Writing – original draft, Data curation, Conceptualization. **Masahiro Koyama:** Writing – original draft, Data curation, Conceptualization.

## Ethics approval and consent to participate

This study was approved by the Research Ethics Committee of the Saitama Medical University Hospital (Approval number: 2022–035). All participants provided written informed consent. All experiments were performed in accordance with the relevant guidelines and regulations.

## Funding sources

This work was supported by JSPS KAKENHI (grant number 23K14902).

## Consent for publication

All participants provided written informed consent for publication.

## Declaration of Competing Interest

The authors declare that they have no competing interest.
